# Evidence of Loss of Tumour-specific Antigen on Repeatedly Transplanting a Tumour in the Strain of Origin

**DOI:** 10.1038/bjc.1962.55

**Published:** 1962-09

**Authors:** M. F. A. Woodruff, M. O. Symes


### EVIDENCE OF LOSS OF TUMOUR-SPECIFIC ANTIGEN ON REPEATEDLY TRANSPLANTING A TUMOUR IN THE STRAIN OF ORIGIN

M. F. A. WOODRUFF AND M. O. SYMES

*From the Department of Surgical Science, University of Edinburgh*

Received for publication July 17, 1962

SPLENOMEGALY has often been observed in mice bearing tumour transplants, and can occur even when the tumour originated in an animal isogenic with the recipient. This may sometimes be due, as has been suggested, to associated infection, or to extra-medullary haemopoiesis resulting from tumour-induced anaemia (van Ebbenhorst Tengbergen and Mühlbock, 1958). In experiments reported previously from this laboratory, however, splenomegaly developed regularly in the absence of infection in A-strain mice which received transplants of a spontaneous A-strain mammary carcinoma, or of the same tumour after 1–5 passages each of 2–3 weeks' duration, and as the histological findings were characteristic of immunological stimulation it was concluded that the tumour possessed one or more antigens which were not represented in normal A-strain tissues (Woodruff and Symes, 1962).

Subsequently, a chance observation suggested that after many passages the tumour was no longer capable of eliciting splenomegaly in the A-strain, and the following experiments were designed to investigate the matter.

### MATERIALS AND METHODS

Four similar experiments were performed. In each one tissue from a spontaneous mammary carcinoma which developed in an adult A-strain female mouse was transplanted subcutaneously to the right flank of three adult (17–21 g.) females of the same strain by the technique described previously. Two weeks later the recipients were weighed and killed by neck dislocation, and their tumours, spleens, and axillary and inguinal lymph nodes* were removed. The spleens and lymph nodes were weighed to the nearest mg. and the spleen ratio was calculated by dividing the weight of the spleen in mg. by the weight of the mouse in g. The total nucleated spleen cell count was determined as described previously (Woodruff and Symes, 1962). Sections were cut and stained with haematoxylin and eosin or pyronin-methyl green (Unna-Pappenheim stain). The tumours were examined macroscopically and sections were cut and stained with haematoxylin and eosin. Material from one tumour, chosen at random, was transplanted subcutaneously to three more A-strain adult females. This process was repeated every 2 weeks for up to 10 passages (Fig. 1).

In the third experiment the spleen and lymph nodes were also removed from the animal bearing the primary tumour at the time of the first transplantation, and examined in the manner described.

* In the first experiment the lymph nodes were not removed until the 8th passage of the tumour.

### RESULTS

The spleen ratios, nucleated spleen cell counts, and weights of the ipsilateral and contralateral lymph nodes for the first three experiments are recorded in Table I. The corresponding mean values and standard deviations obtained

FIG. 1.—Scheme of transplantation used for each of the four tumours which have been studied.

for comparison from 10 normal adult A-strain females which had not received tumour transplants, were as follows :—

Spleen ratio : 6·10 ± 0·94
Nucleated spleen cell count (millions) : 156 ± 26·1
Weight lymph nodes (2 axillary + 1 inguinal) on right side (mg.) : 23·9 ± 4·92
Weight lymph nodes (2 axillary + 1 inguinal) on left side (mg.) : 22·7 ± 4·58

It will be seen that in the first experiment the tumour transplants evoked splenomegaly for 6 passages and thereafter failed to do so. At the 10th passage the tumour was left in place for 4 weeks, and was on the point of killing the mouse, but the spleen and lymph nodes were not enlarged. In the second experiment there was splenomegaly, and generalised lymph node enlargement, most marked on the side of the tumour, for the first two passages only.

In the third experiment the tumour caused marked splenic and lymph node enlargement in the mouse in which it arose, but only moderate splenomegaly and no lymph node enlargement when transplanted for the first time. On the other hand in the fourth experiment there has been no falling off in the magnitude of either the splenic or the lymph node enlargement during the 8 transplant generations which have so far been studied.

There was a remarkably close correlation between the weights of the spleen and lymph nodes and the histological findings, a significant increase in weight being invariably associated with evidence of immunological stimulation. This took the form of accumulation of plasma cells, especially in the red pulp of the spleen and the medullary cords of the nodes, and the presence of germinal centres with activated reticulum cells in the Malpighian follicles and, to a lesser extent, in the primary follicles of the nodes (see Woodruff and Symes, 1962, Fig. 6 and 8). Spleens and nodes of normal weight, on the other hand, showed no histological abnormality, except that after the 10th passage in the first experiment one axillary node was completely replaced by metastatic tumour tissue.

### CONCLUSIONS AND DISCUSSION

The present experiments confirm the observations reported previously (Woodruff and Symes, 1962) which led us to conclude that a spontaneous tumour may possess one or more antigens which are not present in the normal tissues of the animal in which it arises. In addition, however, they provide strong evidence that such tumour-specific antigens may be deleted when the tumour is transplanted repeatedly in the strain of origin.

Evidence of antigenic loss in the early transplant generations of transplantable tumours has been put forward previously by Strong (1926), Bittner (1931) and Gorer (1948), but only in relation to tumours transplanted in animals of different genetic constitution to the one in which the tumour arose. It appears from the present experiments that the same phenomenon occurs when this limitation is removed, and it seems likely that antigenic deletion occurs also in tumours which are not transplanted but remain sufficiently long in the animal in which they arise. The particular tumour we have used is not very suitable for investigating the matter because it kills too quickly, but in the third experiment the findings suggest that considerable deletion may have occurred by the time of the first transplantation.

If this hypothesis is correct it would seem to provide an important clue to the understanding of the behaviour of malignant tumours not only in experimental animals but also in man. During the phase of antigenic difference a tumour may conceivably be held in check, or even destroyed, by immunological mechanisms, whereas after deletion of all tumour-specific antigens the natural immunological defences have no *point d'appui* and any restraining effect they may have had is lost. It is at this stage that metastasis might be expected to occur, and the presence of a lymph node metastasis in the first experiment after the 10th passage of the tumour is consistent with this view.

It seems likely that antigenic deletion occurs more readily with some tumours than others. Chemically induced tumours like the fibrosarcoma studied by Koldovský and Svoboda (1962), whose antigenic structure may depend in part on conjugation of the carcinogen with specific host proteins, may well be peculiarly resistant to this change. It should not be assumed however, just because these authors found no evidence of deletion, that this cannot occur with such tumours, since their observations were limited to the effect of only two transplant generations.

It is not suggested that loss of tumour-specific antigen is the only mechanism by which tumours normally escape immunological destruction, for studies with homografts have provided abundant evidence that a tumour may survive and grow in the face of a degree of immunity sufficient to destroy grafts of normal tissues. Moreover, as Koldovský and Svoboda have shown, by producing a sufficiently large amount of antigen, a tumour may induce a state of immunological unresponsiveness in the host and so facilitate its own survival. In so far as antigenic deletion does occur however, this is likely to be of great importance in relation not only to the natural history of cancer but also to the possibility of developing immunological methods of treatment.

SUMMARY

Experiments are described which confirm the observation reported previously from this laboratory that splenomegaly, associated with histological changes characteristic of antigenic stimulation, may occur in A-strain mice bearing spontaneous or transplanted A-strain mammary carcinomas, and show in addition that when this happens there are similar changes in the axillary and inguinal lymph nodes, most marked as a rule on the same side as the tumour. After a certain number of transplant generations, which differs for different tumours, the capacity to evoke these changes in the host is lost, and this is attributed to deletion of specific tumour antigens. It is suggested that such deletion may occur also in tumours which are not transplanted but remain sufficiently long in the animal in which they arise. The implications of this hypothesis are discussed.

This work was supported by a generous grant from the British Empire Cancer Campaign, of which grateful acknowledgement is made. One of us (M. O. S.) is a Medical Research Council Scholar and is indebted to the Council for this support.

We are grateful to Dr. Angus Stuart for guidance in histological matters, and to Mrs. Y. H. S. Slater and Mr. N. Samuel for expert technical help.

### REFERENCES

BITTNER, J. J.—(1931) *Amer. J. Cancer*, **15**, 2202.

VAN EBBENHORST TENGBERGEN, W. J. P. R. AND MÜHLBOCK, O.—(1958) *Brit. J. Cancer*, **12**, 81.

GORER, P. A.—(1948) *Ibid.*, **2**, 103.

KOLDOVSKÝ, P. AND SVOBODA, J.—(1962) *Folia biol., Praha*, **8**, 95.

STRONG, L. C.—(1926) *J. exp. Med.*, **43**, 713.

WOODRUFF, M. F. A. AND SYMES, M. O.—(1962) *Brit. J. Cancer*, **16**, 120.


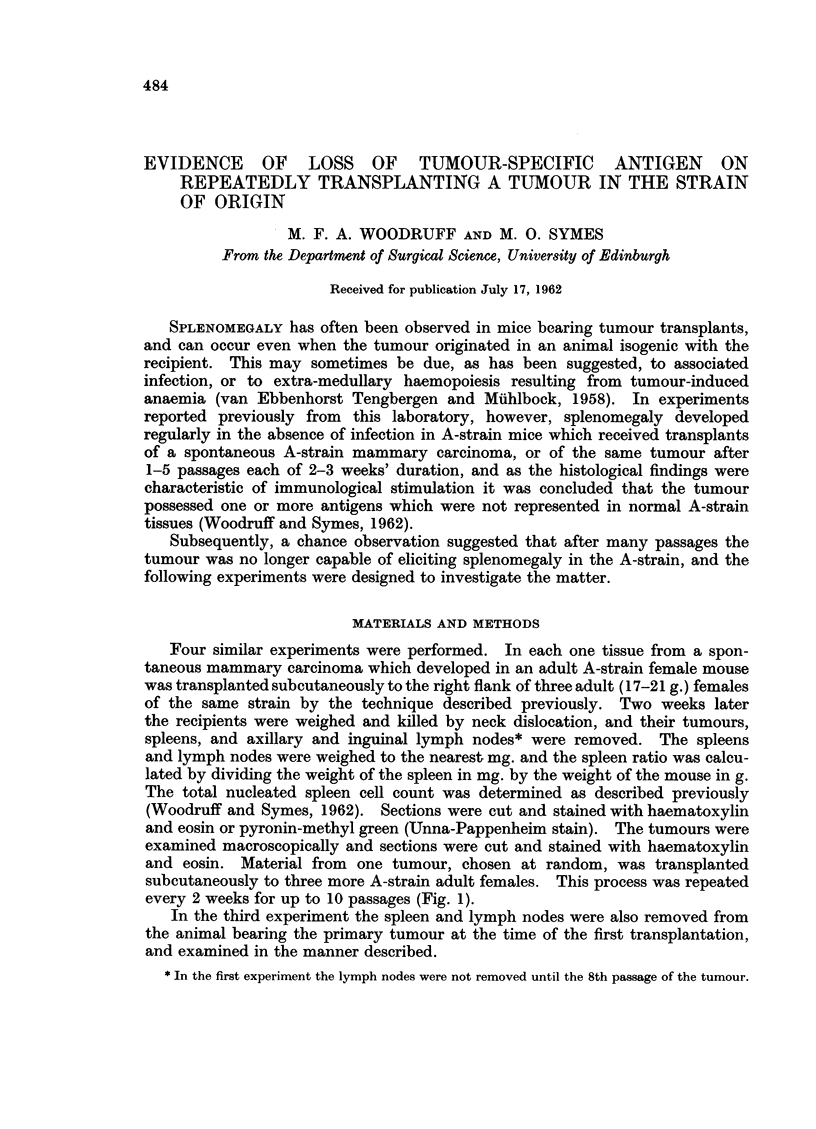

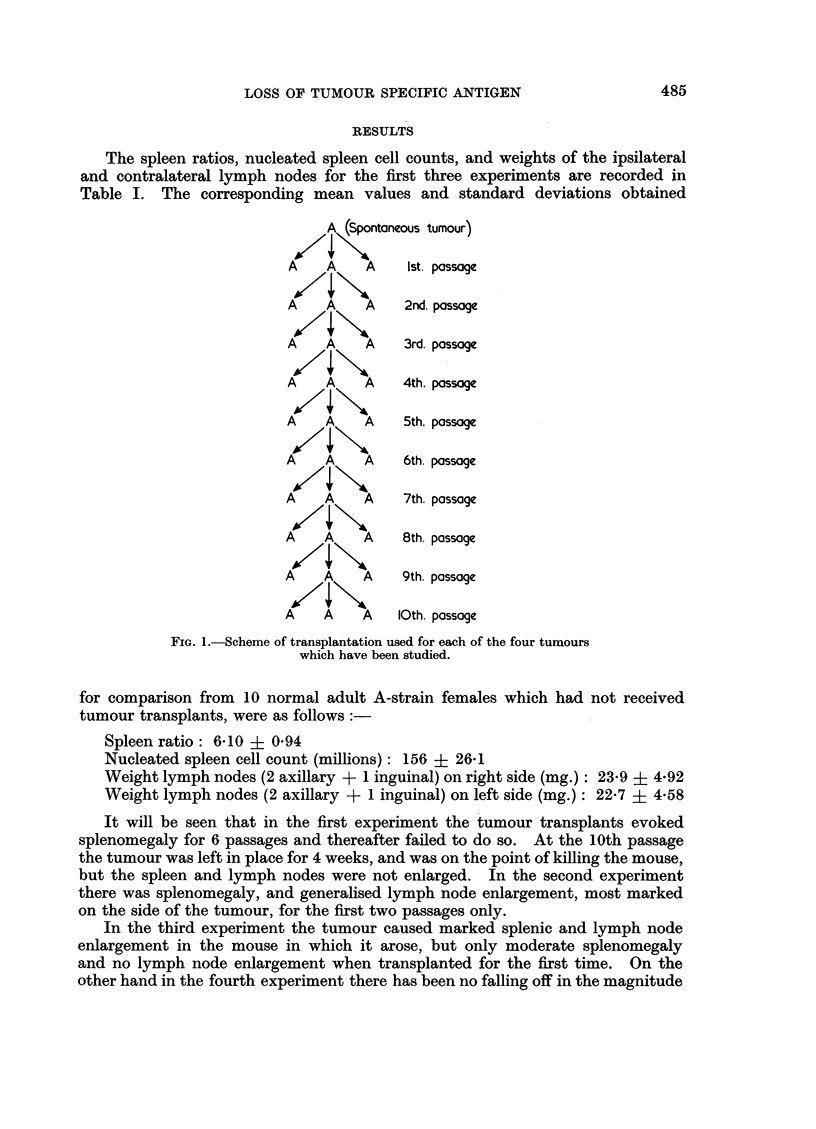

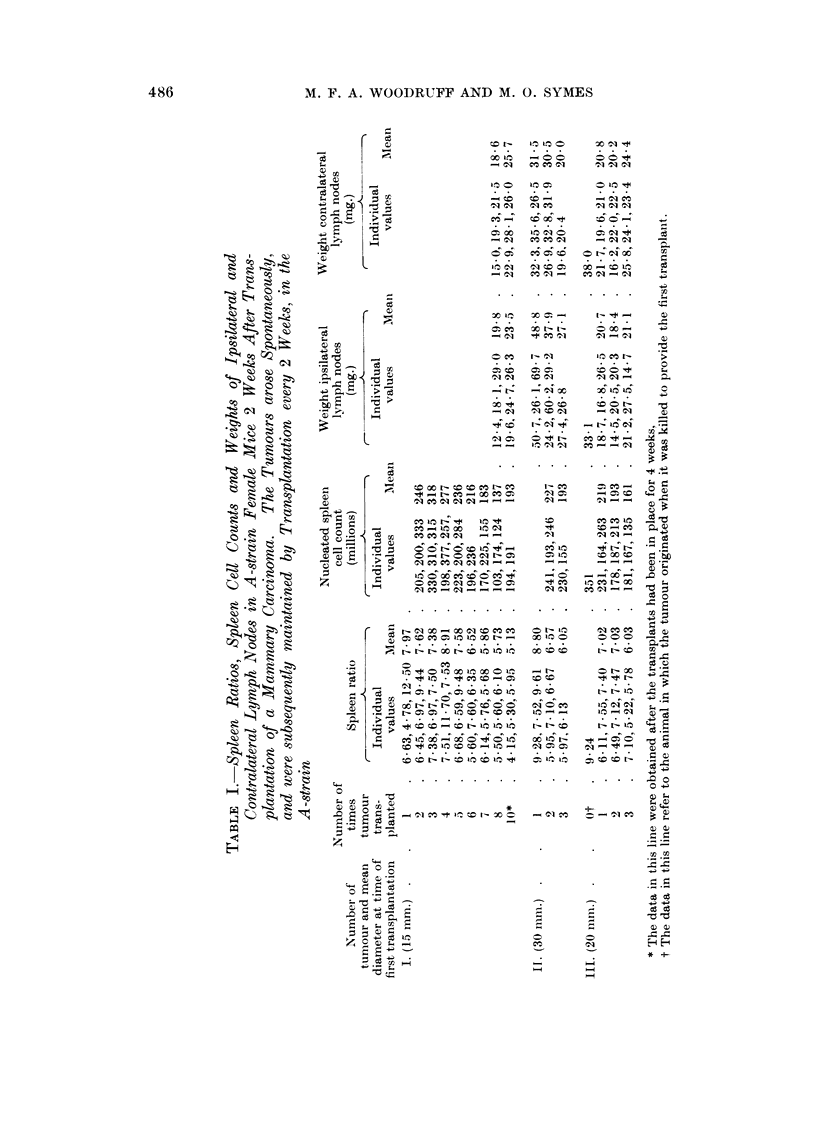

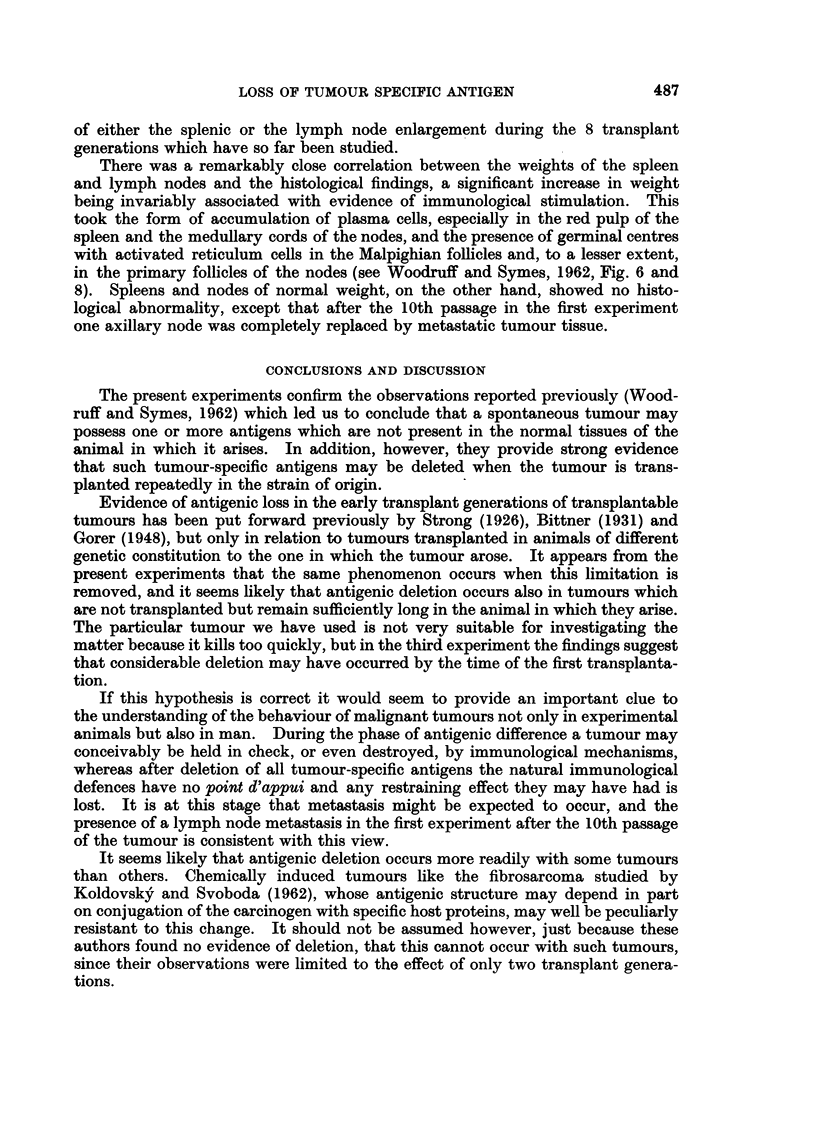

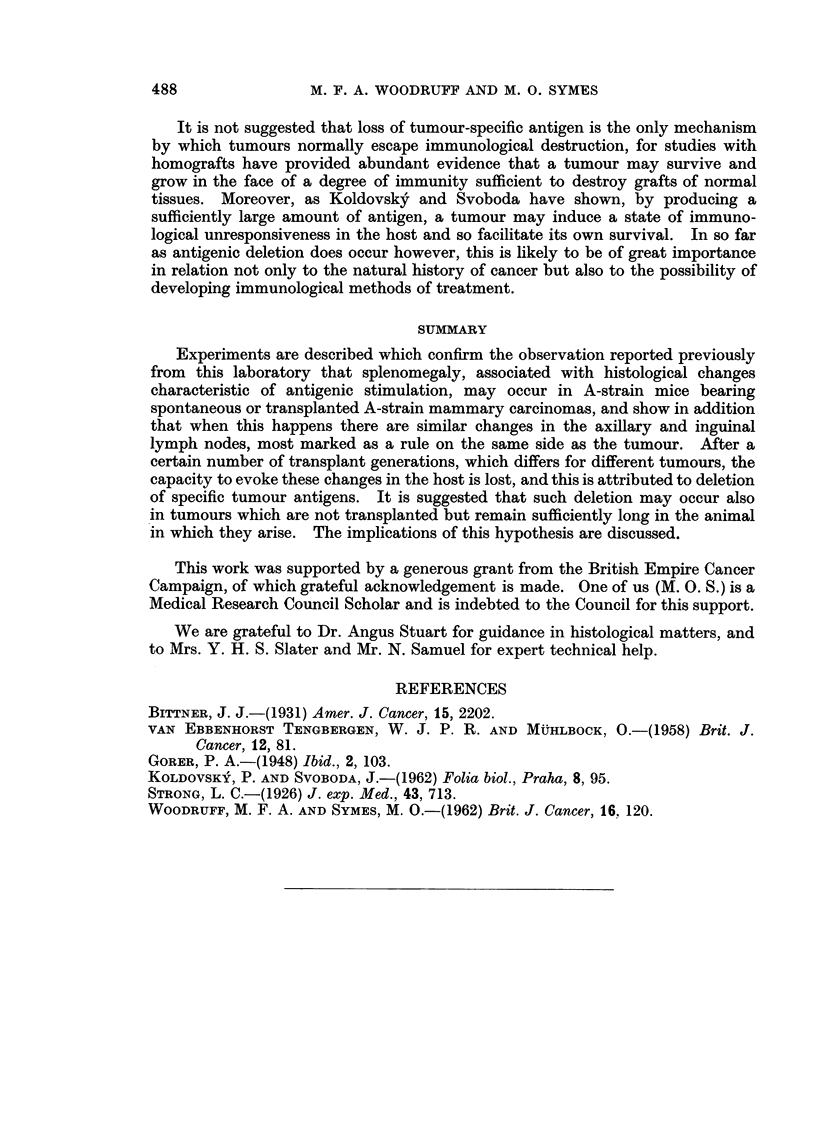


## References

[OCR_00376] WOODRUFF M. F., SYMES M. O. (1962). The significance of splenomegaly in tumour-bearing mice.. Br J Cancer.

